# Surgical Procedures for Planting and Revascularization in Catastrophic Hand Injuries and Preliminary Outcome Assessment: Experience of a Regional Care Center

**DOI:** 10.7759/cureus.99548

**Published:** 2025-12-18

**Authors:** Iram Z González-Vargas, Carlos Regil-Juárez, Daniel Salas-Treviño, Cynthia M González-Cantú, Yanko Castro-Govea

**Affiliations:** 1 Plastic, Aesthetic and Reconstructive Surgery, Hospital Universitario "Dr. José Eleuterio González", Universidad Autónoma de Nuevo León, Monterrey, MEX; 2 Infectious Diseases, Hospital Universitario "Dr. José Eleuterio González", Universidad Autónoma de Nuevo León, Monterrey, MEX; 3 Plastic, Aesthetic and Reconstructive Surgery, Universidad Autónoma de Nuevo León, Monterrey, MEX

**Keywords:** finger reconstruction, hand and microsurgery, hand reconstruction, plastic and reconstructive surgery, revascularization

## Abstract

Replantation and revascularization surgical procedures are a viable option for many patients with devastating upper extremity injuries. Surgical techniques such as microsurgery and rapid multidisciplinary management are crucial in the prognosis of the affected limb. In this study, we present cases of patients with diverse trauma and injury mechanisms that disrupt blood flow to the affected limb. Microsurgical and multidisciplinary approaches were used for quick patient treatment.

In all cases, a rapid initial evaluation was performed, followed by surgical debridement, bone fixation, tendon and nerve repairs, and revascularization procedures using venous and arterial grafts. Immediate results showed successful restoration of vascular flow and preservation of key anatomical structures.

This study demonstrated that complex procedures performed by a multidisciplinary surgical team yielded encouraging results. In addition, the aesthetic, reconstructive, and functional satisfaction of the operated limbs must be evaluated in the long term to establish an objective outcome.

## Introduction

Traumatic injuries in fingers and hands represent a clinical and surgical challenge for plastic surgeons around the world. An estimated 45,000 traumatic finger amputations occur annually in the United States, with laceration, crushing, and avulsion being the primary mechanisms of injury [1].

Since the first successful reimplantation of a thumb [2], advances in microsurgery techniques have made replantation and revascularization a viable option for many patients with devastating upper extremity injuries [2,3]. Reimplantation is defined as the surgical procedure intended to restore the anatomical continuity of an amputated section. Revascularization, on the other hand, is the surgical procedure intended to repair a partially amputated limb through vascular restoration to prevent tissue necrosis [2,4].

The success rate of upper limb injury interventions ranges from 77% to 99% of cases [1-3]. However, revascularization or reimplantation depends on several factors, including ischemia time, injury mechanisms, and patient-specific factors such as age, occupation, and comorbidities [2,5,6].

In this study, we present cases of patients who presented to the emergency department with various types of trauma and injury mechanisms that disrupt blood flow to the affected limb. Microsurgical techniques direct repairs or vascular grafts harvested from distant sites, thereby achieving restoration of blood flow and soft tissue reconstruction at the conclusion of each procedure.

This work intends to demonstrate the surgical procedures and preliminary results obtained from the application of techniques such as microsurgery and organized multidisciplinary management in search of increasing the prognosis of survival of the affected limbs.

## Case presentation

Study design and population

An observational, descriptive, case series study was conducted at the University Hospital “Dr. José Eleuterio González” in Monterrey, México, in the Plastic Surgery Department during the months of March and April 2024. As a protocol, all patients signed informed consent for the performance of the relevant and necessary surgical procedures that were carried out during their hospitalization. Due to the retrospective nature of the study and the adherence to patient confidentiality standards, this study poses no risk to participants, and therefore, authorization from the institutional ethics committee was waived.

All patients treated in the emergency department who sustained upper limb trauma resulting in distal interruption of blood during March and April 2024 and required microsurgical intervention to restore circulation through revascularization or reimplantation were included. Patients with associated trauma compromising life were not included. Also, patients without the amputated segment or if the segment was not preserved under proper cold chain conditions, were discarded from the study; in these cases, amputation was chosen.

The decision to attempt revascularization or reimplantation in the remaining patients was based on multiple factors. First, the patient had to be stable, without hypotension or use of vasopressors. Tissue evaluation was also considered, which was performed in all cases by the hospital's plastic and microsurgery team, ruling out crush or avulsion injuries where blood vessels were visibly damaged. Another factor taken into account was the time since injury (less than six hours), and ensuring that patients with amputated limbs had them properly refrigerated. Finally, comorbidities, level of lesion, extreme contamination, and the opinions of the patient and their family were also considered, as most of these patients were referred from other hospitals to ours with the hope of reimplantation.

The multidisciplinary team that was involved in each case was integrated by emergency medicine, plastic surgery, microsurgery, hand surgery, anesthesiology, psychology, pain management, and physical therapy and rehabilitation. Their roles are described below.

Study procedures

Within the multidisciplinary approach to each patient, the first service to provide care was the shock-trauma emergency service. Once the initial assessment of our patients was completed and advanced trauma management following the ATLS (Advanced Trauma Life Support) protocol was provided, the patients were taken directly to the operating room for a thorough inspection and treatment of the injuries.

The anesthesiology team intervened in each of these patients, administering general anesthesia and a regional brachial plexus block. Since infections are among the most significant negative factors affecting outcomes, all surgical procedures begin with meticulous irrigation and debridement of contaminated, devitalized, and necrotic tissues.

The surgical approach was undertaken by the hospital's microsurgery team, hand surgery team, and plastic surgery team, who were responsible for carrying out the complete surgery. Since infections are among the most significant negative factors affecting outcomes, all surgical procedures begin with meticulous irrigation and debridement of contaminated, devitalized, and necrotic tissues.

Then, they proceeded with the necessary bone fixations to provide skeletal support, after which attention was directed to the remaining structures. This was performed by manual reduction of each of the fractures and subsequent placement of Kirschner wires for fixation, assisted by fluoroscopy.

Nerve and tendon repairs were performed as appropriate. Tendon repairs were of two types, depending on the need: direct repairs with modified Kessler stitches or by using tendon grafts when the gap warranted it. For these, the Pulvertaft technique was used, and the tendons described in each case were used as donors. Prolene 4-0 and 5-0 sutures were used for this.

For nerve repairs, nerve grafts were also used when the gap warranted it, and primary repairs were performed in the remaining cases. The donor nerves were described in each case. In both methods, 9-0 microsurgical sutures were used for coaptations, and the procedures were performed under direct microscope and microsurgical instruments.

Revascularizations and necessary venous repairs were then performed, as with the other tissues. If the gap was significant, venous grafts were used for both repairs, and then anastomoses were performed with 8-0 and 9-0 sutures depending on the caliber of each vessel, using direct vision under a microscope and microsurgical instruments.

Finally, for skin coverage, skin grafts or local flaps were used in cases where necessary. In four of the five cases presented, temporary coverage with a Bogota bag was employed. For this purpose, we used sterile 1,000 cc saline solution bags. These were cut to fit each defect and sutured to the edges to provide temporary coverage. This allowed us to visualize the repairs at all times, was easy to clean by irrigation, did not macerate the repaired tissues, and was easily removed during the final reconstruction. This type of covering is a common tool we use in our hospital due to the characteristics mentioned above. This coverage was used for patients who became unstable before or during surgery, or for patients who had undergone surgeries lasting more than eight hours and still required some repairs or permanent coverage. In these cases, damage control surgery was performed, the most critical tissues were repaired, and temporary coverage was provided. Secondary procedures were done once the patient was in stable condition.

Once each procedure was completed, the patients were evaluated by the pain management service, which prescribed opioid and nonsteroidal anti-inflammatory drug (NSAID) medications to ensure an immediate pain-free recovery. The hospital's psychology team also provided immediate psychological support to each patient and their family. No patient required psychiatric evaluation or medical treatment in this area.

Since bed rest was indicated to protect the performed surgeries, the physical therapy and rehabilitation service initiated lower limb mobilization therapy in bed to avoid complications derived from lack of movement.

This study only shows the immediate results of the first surgery, i.e., the limb-sparing surgery of the injured limb. It is necessary to follow up with these patients in the long term to present the results after rehabilitation therapy to assess the final result and patient satisfaction.

Case presentation

In this case series, the patients treated ranged in age from 15 to 43 years. All the patients were males (a female was dismissed for carrying her amputated finger without the necessary cold chain and for the tissues being macerated from carrying it in cold water for more than two hours), and the most frequently treated extremity was the left (four out of five). None of the operated patients presented any metabolic comorbidity, infectious disease, or adjacent psychological condition; likewise, none reported tobacco use. Injuries were reported at the level of the hand (2), forearm (2), and fingers (1), including flexor zones 1-5 and extensor zones 1-8. Three of the five cases reported complete injuries (vascular, tendons, bones, and nerves) of the extremity in question. Revascularization was the most frequently used technique for salvage of structures, and the most frequent interventions were bone fixation with Kirschner wires, the use of tendon grafts, and the use of flaps for lesion coverage (Table 1).

**Table 1:** Demographic, clinical/diagnostic, and treatment data of the study cases. M = masculine biological sex; h = hours; R = right upper limb; L = left upper limb; X = presence of injury and disruption of 100% of the continuity of the tissue; K wires = Kirschner wires; ALT = anterolateral thigh flap; Tendon graft = using a part of another tendon to repair the damaged one. Tissues injuries: V = arterial/vascular; T = tendon; B = bone; N = nerve. Tamai zones: Zone I: distal to the insertion of the flexor digitorum profundus (FDP); Zone II: interphalangeal joint distal to the insertion of the FDP; Zone III: base of the intermediate phalanx to the distal interphalangeal joint; Zone IV: base of the proximal phalanx to the insertion of the FDS. Zone V: metacarpophalangeal joint and proximal to it.

Sex	Age (years	Level/zone	Ischemia time (h)	Mechanism	Tamai zone	Tissue injury	Number of anastomoses	First surgery	Secondary surgery
M	17	Hand/flexor zones: 2, 3, 4. Extensor zones: 4, 5, 6	2	Crush	4, 5	V, T, B	Arterial: 3, two of them through a venous graft, the rest end-to-end. Venous: 6, end-to-end	K wires, tendon grafts/tenodesis, revascularization, vascular grafts, skin graft	No
M	43	Hand/flexor zone: 3. Extensor zone: 6	2.5	Crush	5	V, T, B, N	Arterial: 7, all through a venous graft. Venous: 2, end-to-end	K wires, tendon repair, tendon graft/tenodesis, revascularization, vascular grafts, local flaps, temporary coverage (sterile bag)	Tendon graft/tenodesis (for flexor pollicis longus repair, donor tendon: plantar flexor tendon of the right foot). Sural nerve graft for median and ulnar nerve repair. ALT free flap for coverage (3 days after first surgery)
M	28	Forearm/flexor zone: 5. Extensor zones: 7, 8	3.5	Cut, avulsion	5	V, T, B	Arterial: 2, end-to-end. Venous: 0	K wires, tendon repair, tendon graft/tenodesis, revascularization, local flaps, temporary coverage (sterile bag)	ALT free flap (5 days after first surgery)
M	40	Forearm/flexor zone: 5. Extensor zone: 7	4 (hand in plastic bag, cold chain conserved)	Guillotine	5	V, T, B, N	Arterial: 2, end-to-end. Venous: 3, end-to-end	K wires, tendon repair, nerve repair, nerve grafts, replantation, temporary coverage (sterile bag)	Extensor tendon repair, ALT free flap for coverage (4 days after surgery)
M	15	Fingers/flexor zones: 1, 2. Extensor zones: 1, 2, 3, 4	3	Avulsion	2, 3, 4	V, T, B, N	Arterial: 1. Venous: 0	K wires, tendon repair, nerve repair, revascularization, skin grafts, local flaps	No

Case 1

**Figure 1:**
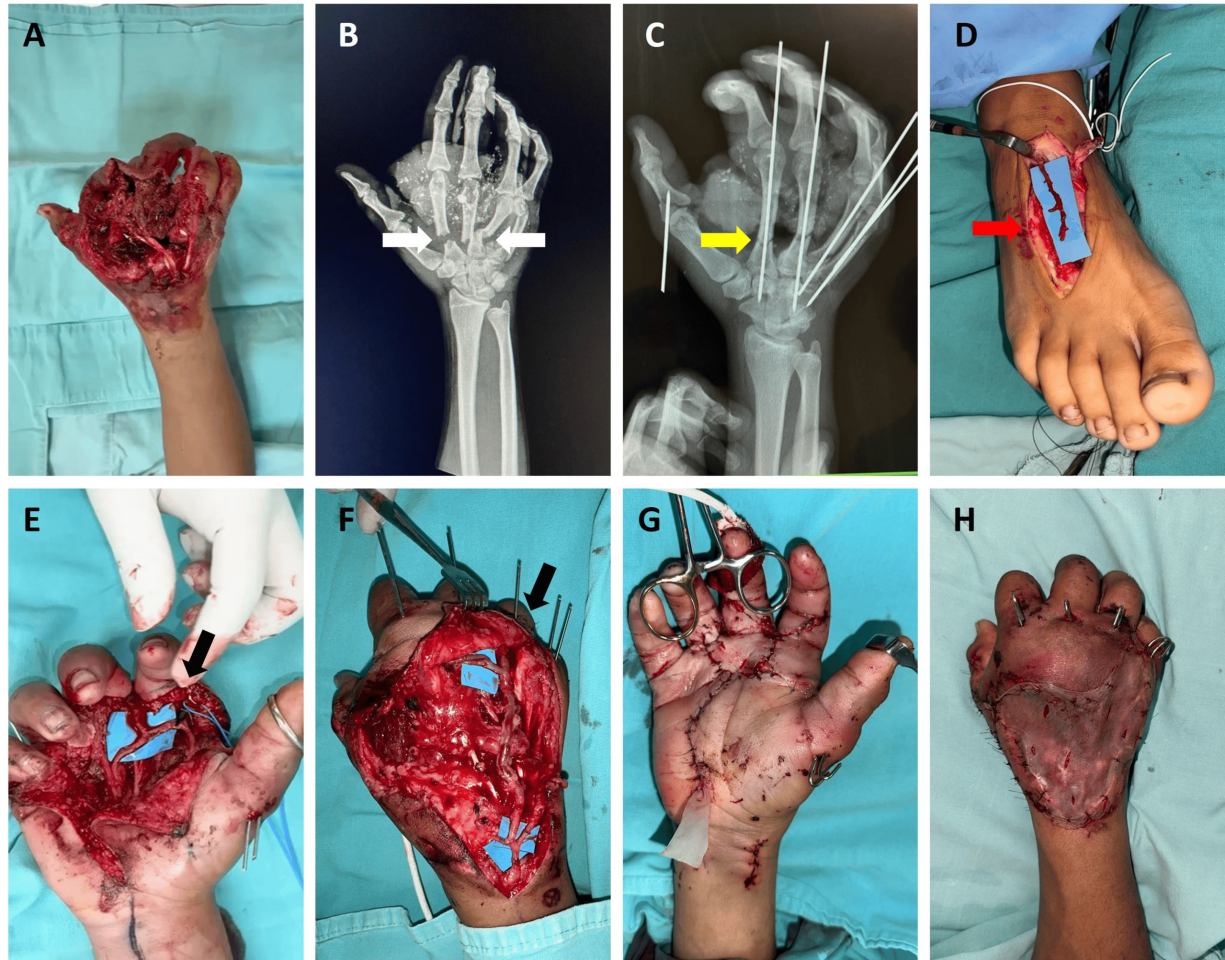
Pre- and post-surgery clinical and X-ray images and treatments in the initial surgery (case 1). (A) Preoperative photograph. (B) Preoperative X-ray with metacarpal fractures (white arrows). (C) Fracture fixation (yellow arrow). (D-F) Harvesting (red and black arrows). (G) Volar closure. (H) Dorsal skin graft.

A 17-year-old male patient presented at the clinic with a crush injury in the right hand. The lesion had bone and tendon exposure, an 8 cm palmar wound involving flexor zones 2, 3, and 4, and extensor zones 4, 5, and 6 (Tamai zones 4, 5), limited digital movement, and absence of blood flow in the third finger (Figure 1A). Imaging revealed fractures of the second to the fifth metacarpals and the proximal phalanx (Figure 1B). Exploration with Doppler ultrasound showed the second finger with filiform flow and no flow in the third finger. The rest of the hand was unremarkable.

Surgical intervention included fracture fixation using Kirschner wires (Figure 1C). Injuries to the common extensor tendons of the second and third fingers were identified and repaired with tendon grafts (using a part of another tendon to repair the damaged one). A venous graft was harvested from the dorsum of the foot (Figure 1D), and a vascular reanastomosis was performed: an arterial anastomosis between the radial interdigital artery of the index and middle fingers, along with six venous anastomoses (Figures 1E, 1F). The palmar wound was closed, and a skin graft was applied to the dorsal aspect (Figures 1G, 1H).

Case 2

**Figure 2:**
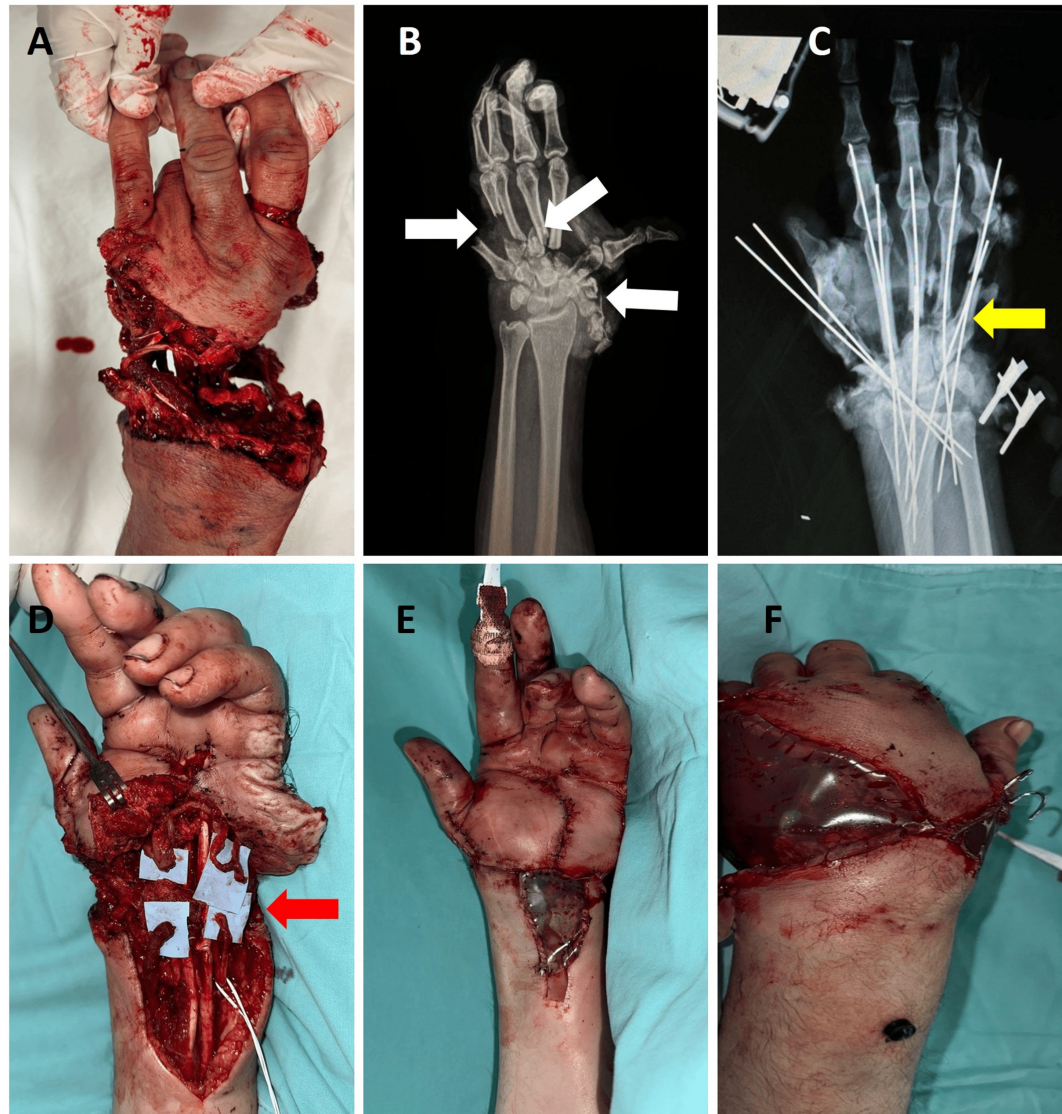
Pre- and post-surgery clinical and X-ray images and treatments in the initial surgery (case 2). (A) Preoperative photograph. (B) Preoperative X-ray with metacarpal and carpal fractures (white arrows). (C) Fracture fixation (yellow arrow). (D) Tendon repair and revascularization of the palmar arch (red arrow). (E and F) Volar wound closure with Bogotá bag.

A 43-year-old male patient presented with a crush injury to the left hand. On examination, there was circumferential avulsion of the soft tissues of the left hand, including flexor zone 3 and extensor zone 6 (Tamai zone 5). At the wrist, bone exposure and fractures were observed, with tendon exposure and complete vascular injury, with total loss of finger mobility, as well as loss of wrist flexion and extension (Figures 2A, 2B).

Surgical treatment included stabilization of the bone structures with Kirschner wire fixation of multiple fractures in the distal carpal row and the metacarpal bases (Figure 2C). Extensive loss and detachment of multiple flexor and extensor tendons were confirmed. For this, transfers of the extensor pollicis brevis (EPB) and extensor pollicis longus (EPL) were carried out, along with plication of the flexor digitorum profundus (FDP) of the fifth finger. Wound reduction and dissection of the radial and ulnar arteries were performed. A saphenous vein graft was harvested from the left lower limb, and palmar arch revascularization was carried out, with anastomoses to the radial, ulnar, and digital arteries (Figure 2D), achieving adequate perfusion to the fingers, confirmed by capillary refill and Doppler ultrasonography. Two venous anastomoses were also performed using dorsal hand veins.

Finally, soft tissue closure was performed using skin flaps, and a sterile bag was applied for temporary coverage due to the high number of hours of surgery and the inherent need for coverage with a free flap and nerve repairs (Figures 2E, 2F).

Case 3

**Figure 3:**
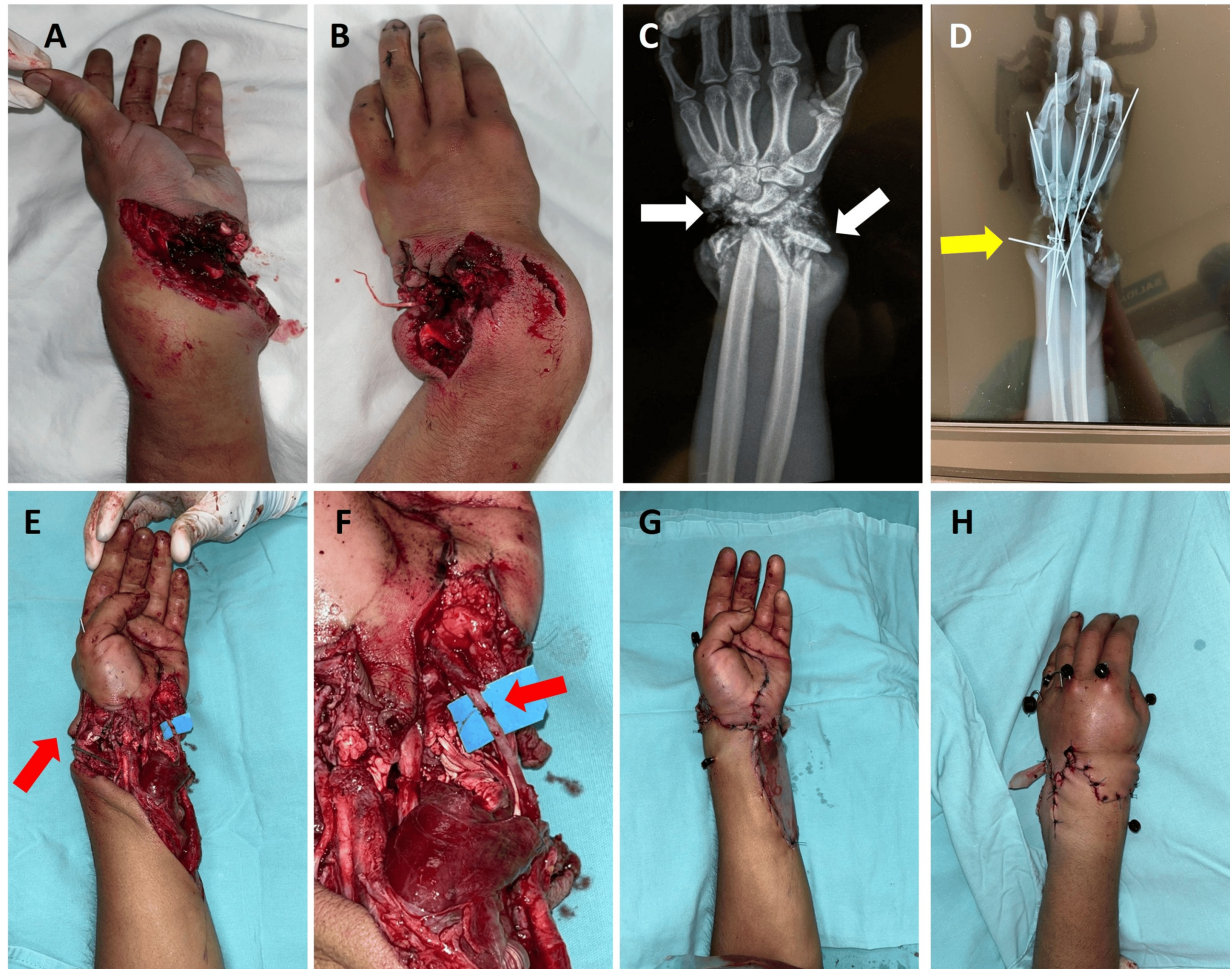
Pre- and post-surgery clinical and X-ray images and treatments in the initial surgery (case 3). (A and B) Preoperative volar and dorsal photograph. (C) Preoperative X-ray with carpal and distal forearm fractures (white arrows). (D) Fracture fixation (yellow arrow). (E and F) Radial and ulnar revascularization (red arrows). (G) Volar wound closure. (H) Dorsal wound closure.

A 28-year-old male patient with a five-year history of occasional marijuana use presented with a machete-related injury to the left hand, involving flexor zone 5 and extensor zones 7 and 8 (Tamai zone 5). On physical examination, the left upper limb at the wrist level showed an avulsion injury, open multi-fragmentary fractures of the ulna and radius, mild bleeding, absent radial and ulnar pulses, and capillary refill >5 seconds (Figures 3A, 3B). Flexion and extension movements were absent in all five fingers, with complete loss of function of the flexor digitorum superficialis (FDS) and FDP, and reduced sensation in all five digits.

During surgery, bone loss was noted in the distal region of the radius and ulna, along with fragments of carpal bones (Figure 3C). Arthrodesis of the radius and ulna to the carpal bones was performed using Kirschner wire fixation (Figure 3D). Subsequently, revascularization of the radial and ulnar arteries was carried out, before the rest of the repairs, due to the long ischemia time and because both the radial and ulnar arteries were completely sectioned, and the hand had no blood flow.

Tendon transfer of the palmaris longus to the flexor pollicis longus (FPL) was performed, along with tenorrhaphy of the flexor carpi radialis (FCR) and the extensor digitorum profundus (EDP) of the third finger (Figures 3E, 3F). Avulsion of the FDS of the fourth and fifth fingers was observed from its origin. The dorsal wound was closed, and a sterile bag was applied at the anterior aspect for temporary coverage due to the high number of hours of surgery and the inherent need for coverage with a free flap, in addition to being hypotensive (Figures 3G, 3H).

Case 4

A 40-year-old male patient with no relevant medical history presented to the emergency room with a traumatic amputation at the distal radius with active bleeding controlled with a tourniquet. The lesion involved flexor zone 5 and extensor zone 7 (Tamai zone 5).

**Figure 4:**
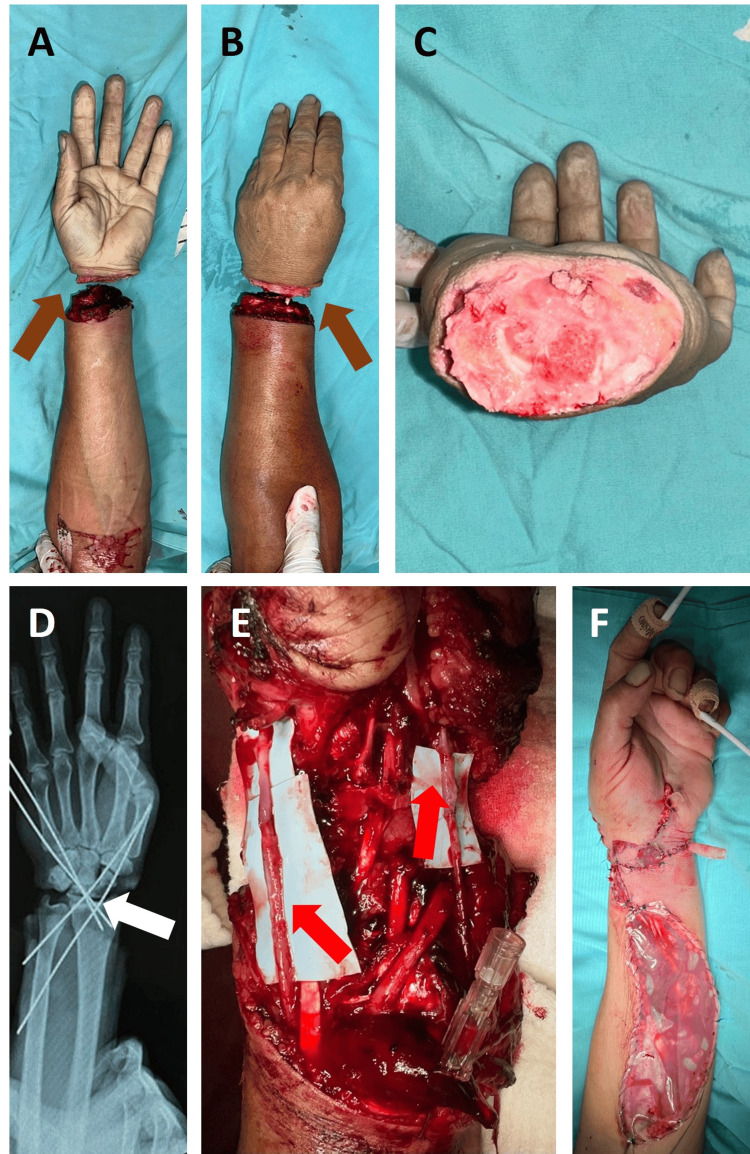
Pre- and post-surgery clinical and X-ray images and treatments in the initial surgery (case 4). (A and B) Preoperative volar and dorsal photograph with total amputation (brown arrows). (C) Complete hand amputation. (D) Fixation of the carpus to the radius and ulna (white arrow). (E) Radial and ulnar revascularization (red arrows). (F) Wound closure and Bogotá bag.

Intraoperatively, a complete amputation of the left upper extremity at the level of the wrist joint was confirmed (Figures 4A, 4B). Wound debridement and exploration were performed (Figure 4C). Kirschner wire fixation was used to stabilize the bone, from the radius to the bases of the fourth and fifth metacarpal, with adequate fluoroscopic control (Figure 4D).

Repair of the FDP tendons was carried out. In this case, it was decided to repair the flexor tendons first due to the need to quickly perform arterial anastomoses in the flexor region to restore blood flow to the hand; the extensor tendons were repaired in a second procedure. Subsequently, the end-to-end anastomosis of the radial and ulnar arteries was performed (Figure 4E). Nerve coaptation was performed using two cable grafts, as well as end-to-end coaptation of the radial nerve.

End-to-end venous anastomoses of three dorsal veins were also performed once the repairs to the flexor region of the hand were completed and the hand was turned over. Vascular patency was confirmed by capillary refill and Doppler ultrasonography. The procedure ended with hemostasis and wound closure. A sterile bag was applied at the anterior aspect for temporary coverage after performing fasciotomies to prevent compartment syndrome due to the high number of hours of surgery, the need for extensor tendons repair, and the inherent need for coverage with a free flap (Figure 4F).

Case 5

**Figure 5:**
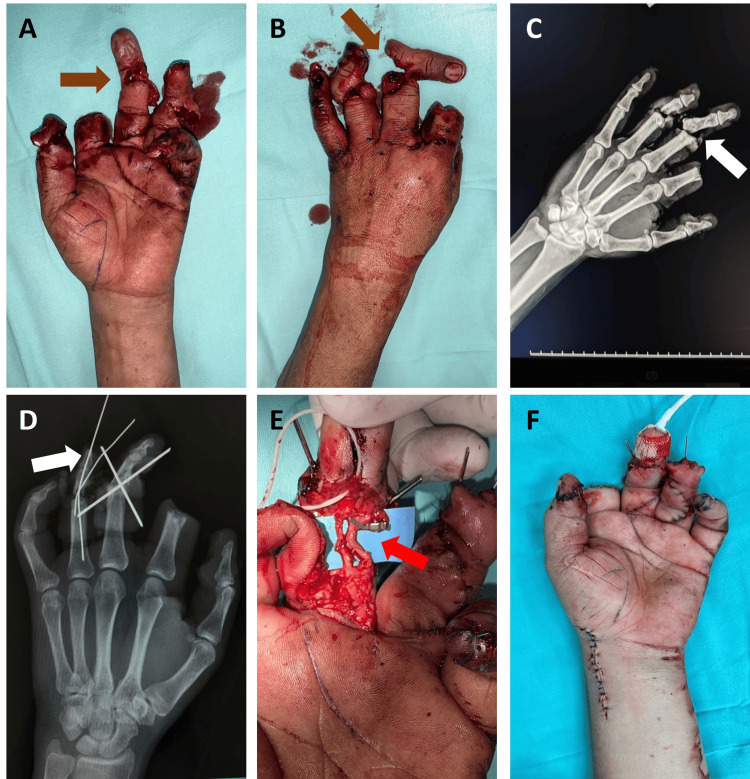
Pre- and post-surgery clinical and X-ray images and treatments in the initial surgery (case 5). (A and B) Preoperative volar and dorsal photograph, multiple wounds (brown arrows). (C) Preoperative X-ray with phalangeal fractures (white arrow). (D) Fracture fixation (white arrow). (E) Third finger revascularization (red arrow). (F) Wound closure.

A 15-year-old male patient, with no relevant medical history, sustained an injury to the left hand caused by an electric saw. On physical examination, the left hand showed multiple wounds with irregular borders, macerated tissues, the first finger with pulp loss, the second finger with amputation at the level of the proximal phalanx, the third and fourth fingers with partial amputation, which remain connected by a skin bridge, the third finger without capillary refill, and the fifth finger with a distal tip injury, without bone exposure. All injuries were found in flexor zones 1 and 2, and extensor zones 1, 2, 3, and 4 (Tamai zones 2, 3, and 4) (Figures 5A-5C).

Surgical debridement of the wounds was performed. In the fourth finger, bone tissue loss was observed in the middle phalanx, and the articular surface of the phalanx was removed. Arthrodesis with Kirschner wires was carried out (Figure 5D). Tendon repair of the extensor and FDS was performed, followed by wound closure with a local advancement flap and placement of a nail splint, showing adequate capillary refill. In the third finger, arthrodesis of the proximal interphalangeal joint was done using Kirschner wires, as well as tenorrhaphy of the extensor, FDS, and FDP tendons.

In addition, an end-to-end anastomosis of the ulnar digital artery and neurorrhaphy of the ulnar digital nerve were performed (Figure 5E). The second finger had a total amputation at the proximal interphalangeal joint level with no available tissue for reconstruction, so a stump remodeling was done. The first finger showed a raw area on the pulp with viable fatty tissue and no exposure of deep structures, and was covered with a partial-thickness skin graft taken from the medial aspect of the ipsilateral arm. The procedure concluded with adequate capillary refill in the treated fingers and Doppler ultrasonography (Figure 5F).

## Discussion

Traumatic hand injuries represent a surgical emergency, especially when they involve complete amputation or complex vascular lesions with severe functional implications. In this case series, we present five cases of replantation and revascularization of the upper extremity treated with microsurgical techniques aimed at preserving upper limb functionality.

Studies have shown that when deciding to perform microsurgery for revascularization or reimplantation of an upper extremity segment, the following sequence should be carried out to achieve the best possible outcome. This was the sequence followed in the cases presented: identification of structures, irrigation and debridement, bone fixation, extensor tendon repair, flexor tendon repair, arterial anastomosis (which, depending on the timing, may need to be performed after bone fixation), release of potential triggers of compartment syndrome, nerve repair, venous anastomosis, and soft tissue coverage [4,7,8]. This sequence was followed in the cases reviewed above, with the order modified only in those cases where blood perfusion was urgent due to the duration of ischemia. Final coverage with temporary methods, such as a sterile bag, was used in cases where a second procedure was necessary, either due to prolonged surgical time or patient instability.

It is important to note that the success of these interventions should not be evaluated solely from a surgical standpoint. The ultimate object of treatment is for the patient to recover a functional status as close as aesthetically acceptable, which would constitute a successful reimplantation outcome [3,7-9]. In our study, the reported cases achieved restored vascular flow; nevertheless, long-term functional follow-up is necessary.

Moreover, several studies emphasize the importance and frequency of secondary surgeries following replantation or revascularization, which raises the need to include these aspects in treatment planning and informed patient consent [5,8,9]. In the specific context of this study, three of the five cases presented received surgeries to provide definitive soft tissue coverage through free flaps once the patients were stabilized, three to five days after the initial surgery, or to complete certain repairs that could not be performed during the initial surgery due to the patient’s instability.

In specific cases, microsurgery is the necessary and sometimes the only tool for revascularization or replantation of upper limb segments [4]. Absolute criteria for replantation or revascularization have been described, such as thumb injuries in children, and injuries to the palm, as well as relative criteria, including ring avulsions, distal or fingertip amputations, extreme wound contamination, and trauma mechanisms such as crush injuries or injuries at multiple levels [4]. However, once these criteria are met, the absolute contraindication includes destructive crush injuries, multi-level destructive amputations, life-threatening injuries, severe concomitant diseases, prolonged ischemia, psychiatric conditions, alcoholism, peripheral vascular disorders, or other conditions leading to prolonged ischemia [3,4]. In the specific case of the patients operated on in this study, both absolute and relative criteria described in the literature were met, and none presented absolute contraindications.

However, it is important to mention that the approach to these cases must be multidisciplinary, considering reconstructive, psychological, and rehabilitation factors, among others, with the ultimate goal of achieving the most functional hand or limb possible. Not everything depends solely on the initial surgery, but also on the adequate performance of each specialist involved in each case, and even the patient, to achieve successful results.

As described in this study, a successful reimplantation is defined as a result that is functional over time, aesthetically acceptable to the patient, and, overall, provides satisfaction to each individual. However, this study focuses on presenting the initial surgeries, showcasing highly complex microsurgical procedures that achieved the primary objective of restoring blood flow to each of the operated limbs. While this does not allow us to confirm that these reimplantations will ultimately be considered successful in follow-up, we can guarantee that achieving this success requires an initial surgery with positive results, such as those presented here, where the main structures were repaired and subsequently rehabilitated to achieve the best possible outcome. The main limitation of this study, therefore, is the need to follow up with each of these patients to present long-term results in a subsequent study after secondary procedures and rehabilitation therapy.

Each of these patients will be monitored by all the services involved, receiving the necessary surgical, medical, and rehabilitation treatments to evaluate the functionality of each of these limbs within an appropriate timeframe to complement this study in the future.

## Conclusions

In summary, these cases demonstrate that, with a trained surgical team and adequate resources, complex procedures can be performed with encouraging results. Following the appropriate path from the initial assessment, analyzing the patient's condition, the type of trauma, the progression time, the state of the amputated tissues, and considering all the surgical steps mentioned in this study and their correct order are important factors that must be complemented to achieve this goal.

Finally, it is worth mentioning that this work focused exclusively on describing each of the surgical techniques employed to achieve an adequate immediate result, restoring blood flow to each limb and saving it immediately, while also performing the necessary repairs to achieve adequate long-term function. However, this study requires prospective follow-up of the cases to collect data on these long-term results, including limb viability and rehabilitation. This series of cases demonstrates technical feasibility and multidisciplinary coordination, but does not prove long-term functional effectiveness.
